# Alpha-Amylase Inhibition and Antioxidative Capacity of Some Antidiabetic Plants Used by the Traditional Healers in Southeastern Nigeria

**DOI:** 10.1155/2017/3592491

**Published:** 2017-03-06

**Authors:** Sunday O. Oyedemi, Blessing O. Oyedemi, Ifeoma I. Ijeh, Princemartins E. Ohanyerem, Roger M. Coopoosamy, Olayinka A. Aiyegoro

**Affiliations:** ^1^Department of Nature Conservation and Ethnobotany, Mangosuthu University of Technology P.O. Box 12363, Jacobs, Durban 4026, South Africa; ^2^Department of Plant Science and Biotechnology, College of Natural Sciences, Michael Okpara University of Agriculture, Umudike, Abia State, Nigeria; ^3^Department of Biochemistry, College of Natural Sciences, Michael Okpara University of Agriculture, Umudike, Abia State, Nigeria; ^4^GI Microbiology and Biotechnology Unit, Agricultural Research Council, Animal Production Institute, Irene, Pretoria 0062, South Africa

## Abstract

Oxidative stress plays a significant role in the pathogenesis of metabolic syndrome including diabetes mellitus (DM). The inhibition of alpha-amylase is an important therapeutic target in the regulation of postprandial increase of blood glucose in diabetic patients. The present study investigated the alpha-amylase inhibitory and antioxidant potential of selected herbal drugs used in the treatment of DM by the traditional healers in Isiala Mbano and Ikwuano regions of southeastern Nigeria. Antioxidant activity was evaluated in terms of free radical scavenging, reducing power, and total phenolic (TPC) and flavonoid content (TFC) in consonance with the TLC profiling. The results showed that methanol crude extracts from* Anacardium occidentale* (AO) and* Ceiba pentandra* (CP) recorded higher TPC and TFC, potent free radical scavenging, and efficient reducing power (RP) as compared with other plant samples. All the plant extracts exhibited a relative alpha-amylase inhibition apart from* Strophanthus hispidus* (SH) extract with a negative effect. We discovered a mild to weak correlation between alpha-amylase inhibition or antioxidative capacity and the total phenol or flavonoid content. At least in part, the results obtained in this work support the traditional use of certain plant species in the treatment of patients with DM.

## 1. Introduction

Type 2 diabetes mellitus (T2DM) is a complex noncommunicable disease associated with pancreatic *β* cell dysfunction and insulin resistance leading to postprandial hyperglycemia [1]. The disease continued to be a global health challenge and economic burden due to modern lifestyle and increased consumption of carbohydrate. The frequency may escalate, with a significant impact on the population of developing countries owing to the absence of efficient and affordable interventions of DM. Under the diabetic condition, chronic hyperglycemia, if not treated, enhances the production of mitochondrial and nonmitochondrial reactive oxygen species (ROS). This phenomenon accelerates the activation of protein kinase C (PKC) isoforms, hexosamine pathway flux, polyol pathway flux, and advanced glycation end products (AGE) involved in the hyperglycaemia-induced oxidative damage [2]. Correspondingly, the increased production of ROS has negative regulation of insulin signaling cascade leading to insulin resistance, *β*-cell dysfunction, impaired glucose tolerance, and mitochondrial dysfunction [2]. The chronic exposure of pancreatic *β*-cells to ROS reduces insulin gene expression and insulin secretion due to its unprecedented low level of antioxidant enzymes [3].

One of the therapeutic targets currently introduced in the management of type 2 DM* is* inhibition of *α*-glucosidase and *α*-amylase to decrease the reabsorption of glucose in the intestine [4]. The alpha-amylase (*α*-1,4-glucan-4-glucanohydrolases) is a prominent secretory product of the pancreas and salivary gland responsible for the initial step in the hydrolysis of complex carbohydrate to a mixture of oligosaccharides and disaccharides in the intestinal mucosa. These sugars are further digested to monosaccharide by the action of alpha-glucosidase. The current alpha-amylase and glucosidase inhibitors in clinical use are associated with side effects such as hypoglycemia, diarrhea, flatulence, and bowel bloating that limit their use in the treatment of diabetes and its complications [5]. There is, therefore, an urgent need to search for complementary and alternative therapies with minimal side effects that can serve as adjunct to the management of DM [6].

In recent times, Nigeria was rated as a country in Africa with the highest number of people (1.7 million) diagnosed with diabetes between the age of 20 and 79 years [7]. The current economic recession, low per capita income, and poorly developed healthcare infrastructure may worsen diabetic condition since the conventional drugs are expensive and often unaffordable by the poor population [8]. Interestingly, Nigeria is a country endowed with biodiversity of medicinal plants that are now gaining relevance in traditional medicine for the management of diverse human diseases including DM. Presently, there is a significant number of both the public citizens and health practitioners depending on herbal drugs compared to scientifically validated proved therapies. These herbal drugs may serve as a potential source of novel molecules for the treatment of diabetes that can represent a more cost-effective treatment, with new prospect of fewer side effects [6]. Dietary consumption of herbals with high antioxidants potentials has shown to exert beneficial effects on pancreatic *β* cells in diabetic condition by delaying or preventing beta cells dysfunction against glucose toxicity [1].

Our recent ethnobotanical survey conducted in Isiala Mbano and Ikwuano local government areas in southeastern part of Nigeria revealed twenty-two plant species commonly used in the management of DM. Despite the acclaimed folkloric use of these plants as an antidiabetic agent, there is a dearth of scientific evidence to substantiate the claim. Some of these botanicals are evaluated for their hypoglycemic activity using an animal model, but there is a paucity of scientific data existing on alpha-amylase inhibition and their antioxidative capacity [9]. Therefore, this study aimed at providing scientific information on the antioxidant and alpha-amylase inhibitory activities of nine plant species grown in the southeast of Nigeria to validate the acclaimed use by the traditional medicine practitioners in the regions.

## 2. Materials and Methods

### 2.1. Study Area

Ikwuano is a local government area (LGA) of Abia state in Nigeria that is situated between 5°26′N and 7°34′E. The area bounded by Ini LGA of Akwa Ibom state by the west and Umuahia by the north. It is an area of 281 km^2^ with a population of 137,993 at the last population census of 2006. The dominant ethnic group is Igbo with farming as the main occupation. The area is popularly known as the food basket of Abia State because of abundant agricultural produce. Isiala Mbano is an LGA of Imo State, Nigeria, situated between 5°42′N and 7°10′E. The altitude is about 152 m above the sea level with a population of 198,736 at the 2006 census. The people of the region practice subsistence farming under local government agencies policy. The inhabitants of both regions use herbal medications either alone or in combination with modern medicine for the treatment of several diseases. The majority of the people in both LGAs are rural dwellers hence the use of plant-based therapies in the treatment of diverse human diseases such as DM which is very common.

### 2.2. Ethnobotanical Survey

The ethnobotanical survey was conducted between March and May 2006 using a well-structured questionnaire administered to the participants with indigenous knowledge of plants utilized in the areas [10]. The set questions contained the diagnosis of DM, the name of botanicals use, methods of preparation, duration of treatment, side effects, and mode of administration. The people interviewed consisted of women and men both married and unmarried at the age of 30 to 65 with little education qualification.

### 2.3. Plant Materials

Nine herbs, namely,* Chlorophora excelsa* (CE; root),* Strophanthus hispidus* (SH; root),* Picralima nitida* (PN; seed),* Persia americana* (PA; seed),* Loranthus micranthus* (LM; leaf),* Ceiba pentandra* (CP; leaf),* Synsepalum dulcificum* (SD; leaf),* Anthocleista djalonensis* (AD; leaf), and* Anacardium occidentale* (AO; leaf) were collected from the field in April, 2016, through traditional healers in Isiala Mbano and Ikwuano LGAs. We identified the plants by their native name and later authenticated by Mr. Ibe Ndukwe of Forestry and Environmental Department, Michael Okpara University of Agriculture. The vouchers specimen (EBUBE 1–9) was prepared and deposited at the College herbarium. The herbal remedies were made by decoction or infusion by soaking 5 g of the plant samples in 1 L of water and 1/2 of a cup is taken orally three times a day for the treatment of a diabetic patient.

### 2.4. Sample Preparation

The leaves of five botanicals were collected, washed with tap water, and air-dried at room temperature for seven days while the roots were oven dried for three days at 40°C. The outer testa of the seeds from* Persia americana* was removed and cut into a small pellet using a kitchen knife and then oven dried for 36 h at 40°C. The dried plant materials were pulverized to a fine powder using an electric blender and stored in an airtight container for further use. Fifteen grams (15 g) of dried powdered materials was extracted with 150 mL of 100% methanol for 48 h on a mechanical shaker (Stuart Scientific Orbital 20.2, SOSI, Essex, UK) and the extracts were filtered using Buchner funnel and Whatman number 1 filter paper. The filtrate was concentrated using a rotary, evaporated at 40°C to recover the solvent, and air-dried in a fume chamber to give a yield ranging from 4.5 to 8 g.

### 2.5. Thin Layer Chromatography (TLC) Profile

Thin layer chromatography is a simple method for analyzing a complex mixture of compounds based on the distance traveled. The plant extracts (1 mg/mL) were dissolved in methanol and spotted on the plate coated with silica gel 60 F 254 as a stationary phase. The slurry was prepared by dissolving 15 g of silica gel 60 F 254 in 30 mL of distilled water and immediately poured into the plate. The plates were air-dried overnight. About 10 *µ*L of the plant extracts was gently loaded on the base of the plate (5 cm above) using the capillary tube. The plates were allowed to develop in chromatographic tanks consisting of three different solvents (mobile phase) chloroform : methanol : acetic acid (5 : 4 : 1) until the solvent front reaches 3/4th of the TLC plate. The TLC plate was removed and allowed to dry; the spots were detected by iodine vapor, and retention factor (*R*_*f*_) was calculated using the following equation: *R*_*f*_  =  distance traveled by components/distance traveled by solvent.

### 2.6. Determination of Total Phenolic Content (TPC)

The total phenolic concentration in these extracts was quantified by the Folin-Ciocalteu reagent (FCR), using the method of Ghaffari et al. [11]. Briefly, 0.2 mL of the plant extract (2 mg/mL) was added to the reaction mixture consisting of 1 mL of 10% v/v FCR and 0.8 mL of Na_2_CO_3_ (0.075 mg/mL) to give a final concentration of 1 mg/mL of each extract. The resulting mixture was incubated at 45°C with shaking for 15 min and the absorbance was measured at 765 nm. A standard curve was prepared by mixing methanol solution of gallic acid (0.2 mL; 0.025–0.400 mg/mL) with 1 mL of 10%, v/v FCR and sodium carbonate (0.8 mL, 0.075 mg/mL). The experiment was carried out in triplicate, and the results were presented as mean values with standard deviation (±SD). The TPC value was expressed as milligrams of gallic acid equivalent (GAE) per g of dried sample. It was calculated using the following formula: *T* = *C* × *V*/*M*, where *T* is the TPC (mg/g) of extract, in GAE; *C* is the concentration of gallic acid from the calibration curve; *V* is the volume of the extract, mL; *M* is the dry weight (g) of the leaf powder from which the extract was obtained.

### 2.7. Determination of Total Flavonoid Content (TFC)

The amount of flavonoids in the plant extracts was determined using the aluminum colorimetric assay method [8]. Briefly, 1 mL of 2% w/v AlCl_3_ prepared in 100% v/v methanol was added to 1 mL of the sample solution. A yellow color formation after incubation at room temperature for one hour measured at 420 nm using an AJI-C03 UV_VIS spectrophotometer. The standard curve for TFC was obtained using quercetin as a standard drug under the same procedure described in TPC determination. The TFC was calculated using the following formula: *T* = *C* × *V*/*M*, where *T* is the TFC (mg/g) of extract in QE; *C* is the concentration of quercetin established from the calibration curve; *V* is the volume of the extract, mL; *M* is the dry weight (g) of the leaf powder from which the extract was obtained. The TFC present in the extracts was calculated as mg/g of quercetin equivalent (QE).

### 2.8. Antioxidant Assays

#### 2.8.1. Determination of Ferric Reducing Antioxidant Power (FRAP)

The ability of plant extracts to reduce ferric (Fe^3+^) to ferrous (Fe^2+^) was evaluated following the method described by Yen and Chen [12] with slight modification. A volume of 0.3 mL of different concentrations (0.025–2 mg/mL) from plant extract, BHT, ascorbic acid, and rutin prepared in distilled water was mixed with reacting mixture consisting of 2.5 mL of 0.2 M phosphate buffer (pH 6.6) and 2.5 mL of K_3_Fe(CN)_6_ (1% w/v). The resulting mixture was incubated at 50°C for 20 min followed by addition of 2.5 mL of TCA (10% w/v). After vigorous shaking, 2.5 mL of the resulting solution was mixed with 2.5 mL of distilled water and 0.5 mL of FeCl_3_ (0.1% w/v) and then incubated at room temperature for 5 min and then measured the absorbance at 700 nm against a blank sample (without extract).

#### 2.8.2. DPPH Radical Scavenging Assay

The free radical scavenging potential of plant extracts was measured in vitro by the 1,1′-diphenyl-1-picrylhydrazyl (DPPH) according to the method by Tariq et al. [13]. The assay experimented by reacting 1.6 mL of 0.135 mM DPPH dissolved in 100% v/v methanol with 0.4 mL of various concentrations (0.0078–2 mg/mL) of methanol crude extracts. The reaction mixture was vortexed thoroughly and left in the dark at room temperature for 30 min. The absorbance of the mixture measured at 517 nm after 2 min. The percentage inhibition of DPPH radical scavenging activity by the plant extracts was calculated as {(Abs_control_ − Abs_sample_)}/(Abs_control_) × 100 where Abs_control_ is the absorbance of DPPH^+^ + methanol; Abs_sample_ is the absorbance of DPPH radical + sample extract/standard. Here, the concentration of the extracts needed to decrease the absorbance of DPPH radical by 50% was calculated. Rutin (Sigma-Aldrich, ≥94%, HPLC grade) at the same working concentrations of the plant extracts was used as reference drug.

#### 2.8.3. ABTS Radical Scavenging Assay

The method of Re et al. [14] was adopted to determine ABTS radical scavenging activity of the plant extracts. The ABTS radical solution was generated by mixing two stock solutions of 7 mM ABTS and 2.4 mM potassium persulphate in the same ratio and allowing the solution to react for 12 h at room temperature in the dark. The resulting solution was diluted with methanol to obtain an absorbance of 0.706 units at 734 nm. A volume of 1 mL of various concentrations (0.0078–2 mg/mL) of the plant extracts reacted with 2.5 mL of ABTS radical solution in the dark for 15 min and later the absorbance was measured. The percentage of inhibition of ABTS radical by the extracts was estimated using the following equation: ABTS radical scavenging activity = {(Abs_control_ − Abs_sample_)}/(Abs_control_) × 100 where Abs_control_ is the absorbance of ABTS radical + methanol; Abs_sample_ is the absorbance of ABTS radical + sample extract/standard.

### 2.9. Test for *α*-Amylase Inhibitory Activity

Porcine pancreatic *α*-amylase (PPA; A05329G191; 1 mL) was dissolved in 9 mL of 20 mM phosphate buffer (pH 6.9) to give 4 Unit/mL solutions. The stock solution of the plant extracts was prepared by dissolving 1 g of the extract in 5 mL of 2% DMSO to give a concentration of 20 mg/mL. Potato starch (0.5% w/v) was dissolved in 20 mM phosphate buffered saline (pH 6.9) and placed in a boiling water bath to get a clear solution. The alpha-amylase inhibition assay was done using the chromogenic non-pre-incubation method adapted from Sigma-Aldrich [15]. Briefly, 40 *μ*L of plant extract, 160 *μ*L of distilled water, and 400 *μ*L of starch solution were mixed in a screw top plastic tube. The reaction started by the addition of 200 *μ*L of the enzyme solution and the tubes were incubated at 25°C for 3 min at room temperature. The enzyme solution was added at 1 min interval from the start of the reaction. Briefly, 200 *μ*L of the mixture was withdrawn into a separate test tube containing 100 *μ*L of DNS color reagent (50.68 g sodium potassium tartrate dissolved in 70 mL of 2 M NaOH with 0.026 mM of 3,5-dinitrosalicylic acid) and placed in a water bath maintained at 85–90°C for 15 min. The mixture in each tube was diluted with 900 mL of distilled water and the absorbance was measured at 540 nm. For each concentration of the extract used, blank incubation was prepared by replacing the enzyme solution with distilled water (200 *μ*L) at the start of the reaction, to correct for the absorbance generated by the plant extract. Control incubations, representing 100% enzyme activity, were carried out in a similar manner by replacing plant extract with 40 *μ*L of 2% DMSO. All the tests were run in triplicate. From the value obtained, the percentage (w/v) of maltose generated was calculated from the equation obtained from the maltose standard calibration curve (0–0.1% w/v maltose). The level of inhibition was calculated as follows: Inhibition (%) = 100  −  % reaction (at *t* = 3 min), where % reaction = mean maltose in sample × 100/mean maltose in control.

### 2.10. Statistical Analysis

Data analysis was done on Microsoft Excel to obtain descriptive statistics. Means values were separated by the Duncan multiple tests using SAS. The different levels of significance were analyzed using one-way analysis of variance (ANOVA). Values were considered significant at *p* < 0.05.

## 3. Results and Discussion

### 3.1. Ethnobotanical Information

The ethnobotanical information gathered revealed a total number of 22 plant species belonging to 17 families commonly used by the inhabitants of Ikwuano and Isiala Mbano LGAs of southeastern Nigeria in the management of DM (Table 1). Ten traditional healers were interviewed while others refused to provide information. It was difficult to collect information from most healers without payment whereas other thought leaking plant information may affect their source of income or disrespect the traditional belief. Some of the plants reported robust antidiabetic potential in the animal model while the majority of the plants lack scientific data to support their acclaimed folkloric use. Table 1 shows the method of herbal preparation, dosage used, and the route of administration for the treatment of diabetic patients by the traditional healers in the region. Apocynaceae (17.4%) top the list of the plant family mostly used followed by Malvaceae (8.7%). The leaf of the plants was widely used (33.3%) either singly or in combination with other plant parts [16]. The preference for the leaf is unknown, but it is likely due to the convenience in collection and less threat or endangerment with regard to other plant parts. Decoction or infusion is often used as a method of herbal preparation in agreement with Appidi et al. [17]. The herbal concoctions were taken orally by the diabetic patients for an extended period with claims of reducing body weakness, the frequency of urination, and disappearance of sugar in the urine with no sign of toxicity. However, there is insufficient scientific information on the biological activities of most plant extracts consumed in the region for DM treatment.

### 3.2. Thin Layer Chromatography Profiling

Herbal medicines and their bioactive compounds such as alkaloids, flavonoids, glycosides, and saponins are recommended in the management of DM and its complications [18]. Alkaloids act as antihyperglycaemic agent via inhibition of alpha-glucosidase and decrease glucose transport through the intestinal epithelium and potentiation of insulin secretion from pancreatic *β* cells [19]. Studies have shown that dietary intake of polyphenols such as flavonoids, phenolic, and tannins-rich food influences peripheral glucose uptake in both insulin and noninsulin sensitive tissues. Saponins are glycosides with the potential to delay glucose transfer from the stomach to the small intestine. In this work, thin layer chromatography (TLC) known as the easiest, cheapest, cost-effective, and easy-to-operate planar chromatographic techniques was adopted to recognize the secondary metabolites present in these botanicals [20]. The principle involves separation of organic compounds on thin layers of adsorbents coated glass by their retention factor (*R*_*f*_). This method provides a clue about the polarity of secondary metabolites to determine the best solvent for bioactive compounds separation in column chromatography. Here, the TLC profiling of nine extracts indicated the presence of diverse bioactive compounds in the plant extracts with good separation. From Table 2, it is clear that mobile phase 1 consisting of chloroform, methanol, and acetic acid in the volume ratio of 5 : 4 : 1 had a relative weak separation as compared with mobile phase II consisting of toluene : ethyl acetate : acetic acid in the same volume ratio. The methanol crude extract of CE placed into the chromatographic tank containing mobile phase II had the highest number of five bands with *R*_*f*_ values of 0.47, 0.62, 0.75, 0.85, and 0.98, followed by PA with 4 bands having *R*_*f*_ values of 0.46, 0.57, 0.69, and 0.96 while other extracts had between 1 and 3 bands (Table 2). CE and AO had a prominent typical band at *R*_*f*_ value of 0.47 and SD and AD at *R*_*f*_ value of 0.813. The plant extracts SH, CP, LM, and AO exhibited a similar band at *R*_*f*_ value of 0.73. The observed TLC profiles provide a characteristic fingerprint of these plants and may perhaps be useful for their identification.

### 3.3. Antioxidant Assays

#### 3.3.1. TPC and TFC Assay

**Figure 1:**
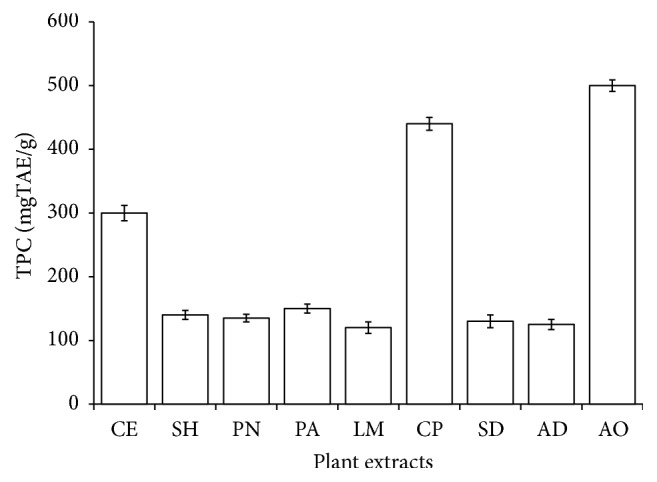
Total phenolic content (TPC) of selected antidiabetic plants used in southeast Nigeria. CE,* Chlorophora excelsa*; CP,* Ceiba pentandra*, SH,* Strophanthus hispidus*, SD,* Synsepalum dulcificum*, PN,* Picralima nitida*, AD,* Anthocleista djalonensis*, PA,* Persea americana*; AO,* Anacardium occidentale;* LM,* Loranthus micranthus*; TAE, tannin acid equivalent.

**Figure 2:**
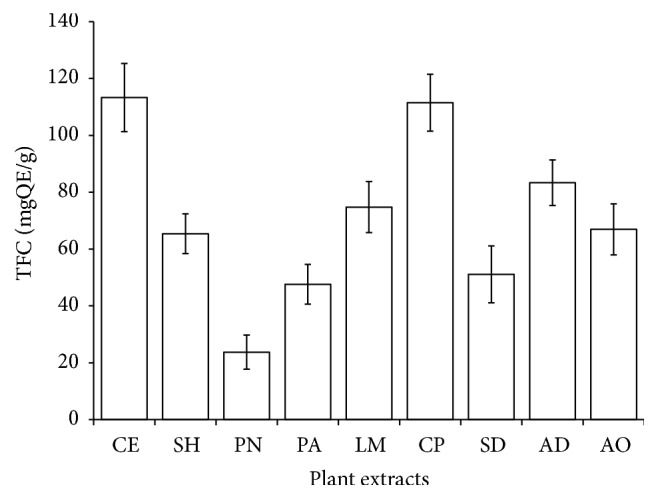
Total flavonoids content (TFC) of selected antidiabetic plants used in southeast Nigeria. CE,* Chlorophora excelsa*; CP,* Ceiba pentandra*; SH,* Strophanthus hispidus*; SD,* Synsepalum dulcificum*; PN,* Picralima nitida*; AD,* Anthocleista djalonensis*; PA,* Persea americana*; AO,* Anacardium occidentale;* LM,* Loranthus micranthus*; QE, Quercetin equivalent.

Plant phenolic and flavonoids compounds such as quercetin, ferulic acid, anthocyanins, catechin, and resveratrol were indicated in epidemiological studies to regulate glycemia via increased glucose uptake, insulin secretion, and inhibition of lipid peroxidation, alpha-glucosidase, and alpha-amylase [21, 22]. These compounds are good H-donating antioxidants that scavenge ROS via chain termination of free radicals depending on the number and position of hydroxyl groups [23]. Epidemiological studies and associated meta-analyses suggested that long-term consumption of diets rich in polyphenols from plant source offer preventive measures against oxidative stress-related diseases [24].  Figure 1 shows the TPC in the plant extracts extrapolated from the standard calibration curve. All the plant extracts recorded a notable amount of phenolic compounds ranging from 100 to 480 mg tannic acid equivalent (TAE)/g. The highest concentration was noted in* Anacardium occidentale* (480 mgTAE/g) followed by* Ceiba pentandra* (420 mgTAE/g) and then* Chlorophora excelsa* (300 mgTAE/g).  Figure 2 depicts the concentration of flavonoids extrapolated from the standard quercetin curve ranging from 23.73 to 113.33 mg quercetin equivalent (QE)/g. The highest flavonoid content of 113.33 mgQE/g was recorded in* Chlorophora excelsa* but the lowest was in* Picralima nitida*. It is obvious that most of these plant extracts are rich in phenolic and flavonoid compounds, which at least in part may support their use in folklore medicine. These compounds are recognized for their significant role in the treatment of free radical stress-related diseases [8].

#### 3.3.2. DPPH Radical Scavenging Activities

**Figure 3:**
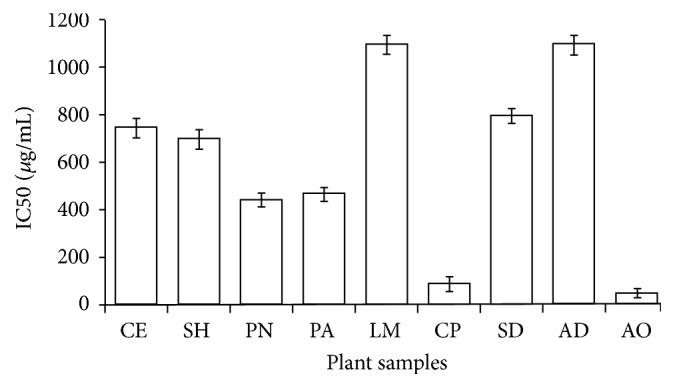
DPPH free radical scavenging activity of methanol crude extracts from selected antidiabetic plants used in southeast Nigeria. CE,* Chlorophora excelsa*; CP,* Ceiba pentandra*; SH,* Strophanthus hispidus*; SD,* Synsepalum dulcificum*; PN,* Picralima nitida*; AD,* Anthocleista djalonensis*; PA,* Persea americana*; AO,* Anacardium occidentale;* LM,* Loranthus micranthus*. Results are expressed as mean ± SD (*n* = 4).

Free radicals such as superoxide anion, hydroxyl radical, oxygen singlet, nitric oxide, and peroxynitrite radicals are chemical species that contain one or more unpaired electron in their outermost atomic orbital. They are responsible for the depletion of immune system, antioxidant, and abnormal gene expression resulting in the etiology of human ailments [25]. All the plant extracts exhibited a considerable DPPH radical scavenging capacity in a concentration-dependent manner having the IC_50_ values ranging from 35 to 1100 *µ*g/mL in the following order: AO > CP > PN > PA > SH > CE > AD > LM (Figure 3). The 50% DPPH radical scavenging effect observed for AO and CP at concentrations of 35 and 50 *µ*g/mL, respectively, is comparable with the standard rutin (IC_50_: 30 *µ*g/mL). The data obtained for AO and CP extracts concurred with that of Fofie et al. [26] and Fazali et al. [27]. There was no significant (*p* < 0.05) correlation between DPPH, TFC (*r* = 0.003), and TPC (*r* = −0.73,) in agreement with the report of Kähkönen et al. [28] who observed that antioxidative capacity of 92 plant extracts did not absolutely depend on their total phenolic content. Often times, the relationship between DPPH, TFC, and TPC depends on their chemical structures, polarities, and solubility in the testing medium [29]. Additionally, Folin-Ciocalteu Reagent measures other components such as sugar, ascorbic acids, and amino acids in the plant extracts which may not give a concise amount of phenolic or flavonoid compounds. Therefore, the observed free radical scavenging activities by the plant extracts suggest a possible synergistic interaction of phenolic compounds with other antioxidants that are not phenolic in nature [30]. The robust antiradical capacities exhibited by CP and AO extracts could play a significant role as complementary or alternative therapy to prevent ROS destruction of pancreatic *β* cells in overt diabetes.

#### 3.3.3. ABTS Radical Scavenging Activities

**Figure 4:**
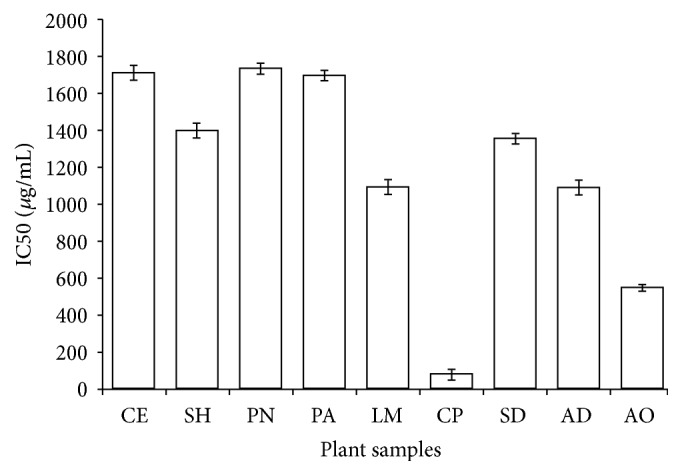
ABTS free radical scavenging activity of methanol crude extracts from selected antidiabetic plants used in southeast Nigeria. CE,* Chlorophora excelsa*; CP,* Ceiba pentandra*; SH,* Strophanthus hispidus*; SD,* Synsepalum dulcificum*; PN,* Picralima nitida*; AD,* Anthocleista djalonensis*; PA,* Persea americana*; AO,* Anacardium occidentale;* LM,* Loranthus micranthus*. Results are expressed as mean ± SD (*n* = 4).

The ABTS radical is a blue chromophore generated by the reaction between ABTS and potassium persulfate having a characteristic absorbance maximum at 734 nm but decreases when reacting with antioxidant [31]. Certain limitations in DPPH assay such as partial ionization of the tested compounds, pH dependence, and lack of radicals of physiological relevance proposed that multiple assays are necessary for antioxidant analysis.  Figure 4 shows the concentration of plant extracts for 50% maximal effect against ABTS radical, ranging from 40 to 1800 *µ*g/mL. All the plant extracts demonstrated an appreciable ABTS radical scavenging effect in this order CP > AO > LM > AD > SD > SH > PA > CE > PN. Here, we observed a moderate correlation between the ABTS and DPPH radicals (*r* = 0.448) which may be related to the stereoselectivity of the radicals with the extracts [32]. It is obvious that DPPH radical scavenging assay seems to be more sensitive than ABTS, which may perhaps be due to the difference of solubility of radicals in the reaction medium. There was a moderate correlation between ABTS radical, TPC (*r* = 0.662), and TFC (0.514). Unfortunately, the methanol crude extract from* Chlorophora excelsa* (CE) exhibited a mild radical scavenging activity despite its richness in TFC and TPC. Interestingly, both CP and AO extracts demonstrated a promising antioxidant potential against ABTS and DPPH radicals contrary to the report of Wang et al. [32]. Our results suggest that CP and AO extracts could be a veritable source of antioxidant that can modulate oxidative stress implicated in the pathogenesis of DM.

#### 3.3.4. Potassium Ferricyanide Reducing Power (PFRP)

**Figure 5:**
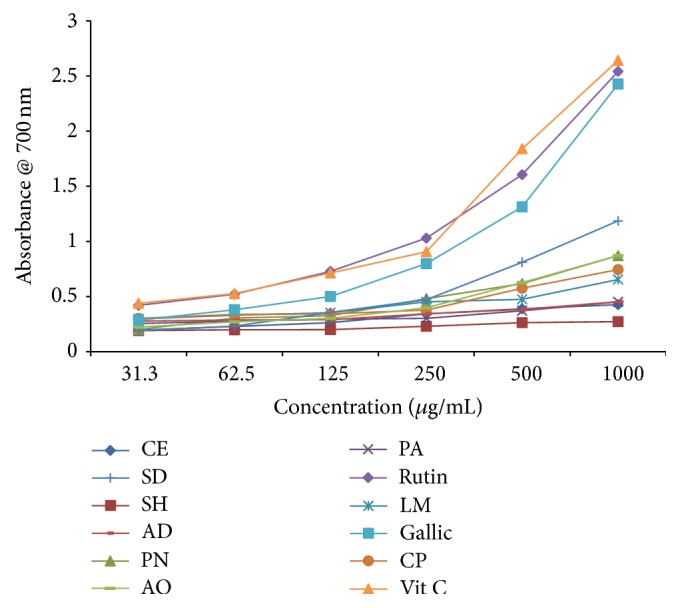
Ferric reducing antioxidant activity of selected antidiabetic plants used in the southeast folklore medicine of Nigeria. CE,* Chlorophora excelsa*; CP,* Ceiba pentandra*; SH,* Strophanthus hispidus*; SD,* Synsepalum dulcificum*; PN,* Picralima nitida*; AD,* Anthocleista djalonensis*; PA,* Persea americana*; AO,* Anacardium occidentale;* LM,* Loranthus micranthus*.

The reducing power (RP) of a compound may serve as a significant indicator of its potential antioxidant capacity to reduce the potassium ferricyanide (Fe^3+^), to form potassium ferrocyanide (Fe^2+^). The formation of ferric ferrocyanide complex after reaction of antioxidant with ferric trichloride measured at an absorption maximum 700 nm [33]. RP is often associated with the presence of reductones that exhibit antioxidant action via chain termination reaction and prevention of peroxide formation [34]. The reducing capacity of the plant extracts was dose dependent in the following order vitamin C > rutin > gallic acid > SD > AO, PN > CP > LM > AD > PA > CE > SH (Figure 5). At 1000 *µ*g/mL, the absorbance values for SD, AO, PN, CP, and LM were 1.186, 0.871, 0.871, 0.744, and 0.654, respectively. The RP of standard ascorbic acid, rutin, and gallic acid was significantly higher than the extracts perhaps due to the complexity of phytochemicals. Most of the extracts displayed strong RP activity though there was no significant correlation found between total phenolic, flavonoids, and reducing power. It is believed that the total number of hydroxyl groups present in the aromatic compounds of herbal products determines its antioxidant capacity [35]. Synergism of these compounds with other components may contribute significantly to the observed antioxidant capacity. It is interesting to note that SD and PN extracts with low concentrations of TPC and TFC exhibited a higher degree of electron donation confirming previous observation in DPPH assay [35]. The present study shows that some plant extracts contain varying concentrations of reductones known to terminate free radical chain reactions that may not be dependent on the total phenolic content. At least in part, the RP values substantiate the ethnomedicinal use of certain herbal therapies in the prevention or treatment of stress-related diseases in man.

#### 3.3.5. Alpha-Amylase Inhibitory Activity

**Figure 6:**
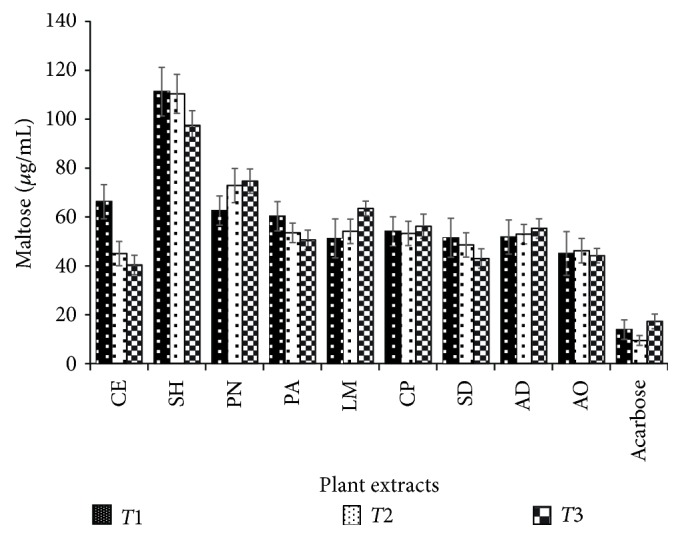
Maltose formation in the presence of selected antidiabetic plants; CE,* Chlorophora excelsa*; CP,* Ceiba pentandra*; SH,* Strophanthus hispidus*; SD,* Synsepalum dulcificum*; PN,* Picralima nitida*; AD,* Anthocleista djalonensis*; PA,* Persea americana*; AO,* Anacardium occidentale;* LM,* Loranthus micranthus* (1 mg/mL). Results are expressed as mean ± SD (*n* = 4).

The inhibition of pancreatic alpha-amylase is one of the therapeutic targets for delaying oligosaccharide digestion to absorbable monosaccharides in the intestinal brush border, resulting in reduced postprandial hyperglycemia [4]. Phenolic compounds such as phenolic acids and flavonoids bind covalently to alpha-amylase and change its activity due to the ability to form quinones or lactones that react with nucleophilic groups on the enzyme molecule [36]. Flavonoids are hydroxylated phenolic compounds having a benzo-*γ*-pyrone structure mostly present in plants in response to microbial infections [37]. Some of these compounds effectively inhibit alpha-amylase activity based on the ability to form quinone with the 4-oxo-pyrane structure of the enzyme via the hydroxyl group at C-3 and C-4 of ring B [38].  Table 3 shows the percent of alpha-amylase (AA) inhibition by the methanol crude extracts. The results showed that CE, SD, AO, PN, PA, AD, LM, and CP extracts (1 mg/mL) were able to inhibit the enzyme by 32.7, 28.4, 26.4, 24.4, 15.6, 7.8, 6.4, and 5.84% at 3 min, respectively. The alpha-amylase inhibition (AAI) was not significant as compared with the standard acarbose (500 *µ*g/mL: 78%) but showed that the extracts contained bioactive compounds that can inhibit AA since less starch converted to maltose. A weak Pearson correlation was observed between AAI and TPC (*r* = 0.25) suggesting a partially formed quinone or lactone due to the steric position of the hydroxyl or methoxyl groups [37, 38]. There was a negative correlation between AAI and TFC signifying that the flavonoids present in the plant extracts did not contribute to AAI [39]. We assumed that these extracts function through other mechanisms as herbal remedies for DM treatment and partly through alpha-amylase inhibition. SH showed a negative result (−62.56%) which gives an impression that the AA is activated rather than inhibited and thus could aggravate DM condition if ingested [38].  Figure 6 showed that AAI of some plant extracts was not time-dependent while that of others increases as the time proceeds.

## 4. Conclusion

We have shown the alpha-amylase inhibition (AAI) and antioxidative capacity of selected medicinal plants used in Ikwuano and Isiala Mbano LGAs in the treatment of DM. Some plant extracts in particular CP and AO exhibited a promising antioxidant potential that can be explored in the development of novel drugs for oxidative stress-related diseases. A weak AAI suggests other possible therapeutic targets for the extracts in the management of DM. Our studies partially confirmed the traditional use of CE, SD, AO, PN, and PA for diabetes management in the region. Further studies are needed to define the precise mechanism of action, bioactive compounds, and curare effect of these botanicals at the dosage recommended by the traditional healers.

## Figures and Tables

**Table 1:** Plants used for the treatment of DM in Ikwuano and Isiala Mbano LGAs in southeast Nigeria.

S/N	Botanical names	Common names	Family name	Plant parts used	Method of preparation
1	*Allium sativum* L	Garlic (C) Aayi (Y) Ayo-Ishi (I) Tafarunna (H)	Amaryllidaceae	Clove	The clove of garlic is boiled and poured into a container and taken orally 3 times a day

2	*Aloe vera* (L) Burm. f.	Ahon-erin (Y) Barbados (C)	Asparagaceae	Leaf	The liquid from the leaves is boiled to powder and soaked in water and 1/2 of a cup of decoction is taken orally

3	*Andrographis paniculata* Wall	King of bitter (C) Mejemeje (Y)	Acanthaceae	Root	The decoction is prepared and taken orally

4	*Anacardium occidentale* (L)	Cashew (C) Kaju (Y) Okpokpo (I) Kanju (H)	Anacardiaceae	Leaf	The leaf of *Anacardium occidentale* is soaked in dry gin

5	*Azadirachta indica* Neem	Dongoyaro (Y) Ogwu (I)	Meliaceae	Leaf	The decoction is made from boiled fresh roots and 2 spoonfuls are taken orally

6	*Anthocleista djalonensis* A. chev	Cabbage (C) Sapo (Y) Akpakoro (I) Putaa (H)	Gentianaceae	Leaf	The leaf of *Anthocleista djalonensis* is boiled and poured into a container

7	*Blighia unijugata* L.	Ako-isin (Y) Okpu ulla (I) Gwanja-kusa (H)	Sapindaceae	Root, bark, leaf	The bark is powdered and infusion of 2 teaspoonfuls are taken orally

8	*Bridelia ferruginea* (Benth)	Ola (I) Iralodan (Y)	Euphorbiaceae	Root, leaf, bark	Fresh leaves are boiled with water and two teaspoonfuls of decoction are taken orally

9	*Catharanthus roseus* (L.) G. Don	Madagascar-Periwinkle (C) Iyere (Y) Oziza (I)	Apocynaceae	Whole plants	The decoction is made from boiled fresh roots and two spoonfuls are taken orally three times a day

10	*Ceiba pentandra* (L) Gaertner	Kapok Araba (Y) Akpu-owu (I) Rimi (H) Silk cotton (C)	Malvaceae	Leaf	The fresh leaf is boiled and 1/2 of a cup is taken orally three times a day

11	*Chlorophora excelsa* (Welw.) Benth	Oji (I)	Moraceae	Root	The root of *Chlorophora excelsa* is cut into sizes and about 3 pieces are soaked in a bottle containing soda water

12	*Costus afer* Ker-Gawl	Okpoto (I) Kakizawa (H) Tete-egun (Y)	Costaceae	Fruits, root, bark	The decoction of the stem or powdered fruits is used in the treatment of diabetes

13	*Gongronema latifolium* (Benth)	Utazi (I) Bush buck (C) Arokeke (Y)	Apocynaceae	Root, leaf, bark	The leaves is infused with hot water and drunk after cooling while the decoction of the root is prepared and taken orally 3 times a day

14	*Ocimum gratissimum* L.	Efinrin (Y) Nchonwu (I) Daidoya (H) Scent leaf (C)	Lamiaceae	Leaf	The infusion of the leaf is prepared and 1/2 of glass cup is taken orally 3 times a day

15	*Loranthus micranthus* Linn.	Ogbuotele egbu nkita (I) Mistletoe (C)	Loranthaceae	Leaf	The leaf of *Loranthus micranthus* is squeezed and the resultant mixture is poured into a container and taken orally

16	*Persea Americana* Mill	Ube bekee (I) Igba (Y) Avocado (C)	Lauraceae	Seed	The seed is crushed and mixed with edible plantain for consumption

17	*Picralima nitida (Stapf) T. Durand and H. Durand*	Otoose (I)	Apocynaceae	Seed	The seed is soaked in a bottle containing soda water overnight and taken orally

18	*Strophanthus hispidus* DC.	Osisikaguru (I)	Apocynaceae	Root	The root is boiled together with the root of *Vernonia amygdalina* and the resulting mixture is poured into a bottle

19	*Synsepalum dulcificum* L.	Agbayun (Y)	Sapotaceae	Leaf	The leaf is squeezed and the resultant mixture is poured into a container and 1/2 glass cup is taken orally.

20	*Theobroma cacao* L.	Cocoa (C)	Malvaceae	Seed	The seed is soaked in a bottle containing soda water overnight and taken orally

21	*Vernonia amygdalina* Delile	Ewuro (Y), Shuwaka (H) Onugbo (I)	Asteraceae	Leaf, root	The decoction of the root or leaf is prepared and 1/2 of a glass cup is taken orally

22	*Zingiber officinale* Roscoe	Atale (Y) Jinga (I) Chita (H) Ginger (C)	Zingiberaceae	Rhizome	Fresh rhizomes are washed and crushed and boiled and 1/2 of a cup of decoction is taken orally

Y: Yoruba; I: Igbo; C: common name; LGAs: local government areas; DM: diabetes mellitus.

**Table 2:** Thin Layer chromatography retention factor using mobile phase C : ME : AA (I) and Tol : EA : AA (II) in the ratio of 5 : 4 : 1.

S/N	Plant samples	*R*_*f*_ values	
		C : ME : AA (I)	Tol : EA : AA (II)
1	*Chlorophora excels*	0.75, 0.97	0.47, 0.63, 0.75, 0.85, 0.98
2	*Strophanthus hispidus*	0.82	0.73, 0.94
3	*Picralima nitida*	0.15, 0.33, 0.70	0.11, 0.21, 0.71
4	*Persea americana*	0.15, 0.82	0.46, 0.57, 0.69, 0.9
5	*Loranthus micranthus*	0.90	0.730
6	*Ceiba pentandra*	0.20, 0.77, 0.9	0.08, 0.70, 0.73
7	*Synsepalum dulcificum*	0.85	0.81
8	*Anthocleista djalonensis*	0.84	0.81
9	*Anacardium occidentale*	0.75	0.47, 0.73, 0.86

C: chloroform; ME: methanol; AA: acetic acid; Tol: toluene; EA: ethyl acetate; *R*_*f*_: retention factor.

**Table 3:** Percentage *α*-amylase inhibition of the selected antidiabetic plants.

Plant extract	Part used	% inhibition
*Chlorophora excels*	Root	32.75
*Strophanthus hispidus*	Root	−62.56
*Picralima nitida*	Seed	24.44
*Persea Americana*	Seed	15.64
*Loranthus micranthus*	Leaf	6.35
*Ceiba pentandra*	Leaf	5.86
*Synsepalum dulcificum*	Leaf	28.35
*Anthocleista djalonensis*	Leaf	7.82
*Anacardium occidentale*	Leaf	26.39

Percentage inhibition was calculated at *t* = 3 min as 100  −  % reaction, whereby the % reaction = (mean maltose in sample/mean maltose in control) × 100.
